# Correction to: The evolution of the ventilatory ratio is a prognostic factor in mechanically ventilated COVID-19 ARDS patients

**DOI:** 10.1186/s13054-021-03849-2

**Published:** 2021-12-17

**Authors:** Antoni Torres, Anna Motos, Jordi Riera, Laia Fernández-Barat, Adrián Ceccato, Raquel Pérez-Arnal, Dario García-Gasulla, Oscar Peñuelas, José Angel Lorente, Alejandro Rodriguez, David de Gonzalo-Calvo, Raquel Almansa, Albert Gabarrús, Rosario Menéndez, Jesús F. Bermejo-Martin, Ricard Ferrer, Rosario Amaya Villar, José M. Añón, Carme Barberà, José Barberán, Aaron Blandino Ortiz, Elena Bustamante-Munguira, Jesús Caballero, Cristina Carbajales, Nieves Carbonell, Mercedes Catalán-González, Cristóbal Galbán, Víctor D. Gumucio-Sanguino, Maria del Carmen de la Torre, Emili Díaz, Ángel Estella, Elena Gallego, José Luis García Garmendia, José Garnacho-Montero, José M. Gómez, Arturo Huerta, Ruth Noemí Jorge García, Ana Loza-Vázquez, Judith Marin-Corral, Amalia Martínez de la Gándara, Ignacio Martínez Varela, Juan López Messa, Guillermo M. Albaiceta, Mariana Andrea Novo, Yhivian Peñasco, Juan Carlos Pozo-Laderas, Pilar Ricart, Inmaculada Salvador-Adell, Angel Sánchez-Miralles, Susana Sancho Chinesta, Lorenzo Socias, Jordi Solé-Violan, Fernando Suares Sipmann, Luis Tamayo Lomas, José Trenado, Ferran Barbé, CIBERESUCICOVID Project (COV20/00110, ISCIII), Berta Adell-Serrano, Alexander Agrifoglio, María Aguilar Cabello, Luciano Aguilera, Victoria Alcaraz-Serrano, Cesar Aldecoa, Cynthia Alegre, Sergio Álvarez, Antonjo Álvarez Ruiz, Rut Andrea, José Ángel, Marta Arrieta, J. Ignacio Ayestarán, Joan Ramon Badia, Mariona Badía, Orville Báez Pravia, Ana Balan Mariño, Begoña Balsera, Laura Barbena, Enric Barbeta, Tommaso Bardi, Patricia Barral Segade, Marta Barroso, José Ángel Berezo García, Judit Bigas, Rafael Blancas, María Luisa Blasco Cortés, María Boado, María Bodi Saera, Neus Bofill, María Teresa Bouza Vieiro, Leticia Bueno, Juan Bustamante-Munguira, Lucia Cachafeiro, David Campi Hermoso, Sandra Campos Fernández, Iosune Cano, Maria Luisa Cantón-Bulnes, Pablo Cardina Fernández, Laura Carrión García, Sula Carvalho, Núria Casacuberta-Barberà, Manuel Castellà, Andrea Castellví, Pedro Castro, Ramon Cicuendez Ávila, Catia Cillóniz, Luisa Clar, Cristina Climent, Jordi Codina, Pamela Conde, Sofía Contreras, María Cruz Martin, Raul de Pablo Sánchez, Diego De Mendoza, Cecilia del Busto Martínez, Yolanda Díaz, María Digna Rivas Vilas, Cristina Dólera Moreno, Irene Dot, Pedro Enríquez Giraudo, Inés Esmorís Arijón, Teresa Farre Monjo, Javier Fernández, Carlos Ferrando, Albert Figueras, Eva Forcadell-Ferreres, Lorena Forcelledo Espina, Nieves Franco, Àngels Furro, Felipe García, Beatriz García, Emilio García Prieto, Carlos García Redruello, Amaia García Sagastume, Maria Luisa Gascón Castillo, Gemma Gomà, Vanesa Gómez Casal, Silvia Gómez, Carmen Gómez Gonzalez, Jessica González, Federico Gordo, Maria Pilar Gracia, Alba Herraiz, Rubén Herrán-Monge, Mercedes Ibarz, Silvia Iglesias, Maria Teresa Janer, Gabriel Jiménez, Mar Juan Díaz, Karsa Kiarostami, Juan I. Lazo Álvarez, Miguel León, Alexandre López-Gavín, Ana López Lago, Desire Macias Guerrero, Nuria Mamolar Herrera, Rafael Mañez Mendiluce, Cecilia L. Mantellini, Gregorio Marco Naya, Pilar Marcos, Enrique Marmol Peis, Paula Martín Vicente, María Martínez, Carmen Eulalia Martínez Fernández, Maria Dolores Martínez Juan, Juan Fernando Masa Jimenez, Joan Ramon Masclans, Emilio Maseda, Eva María Menor Fernández, Mar Miralbés, Josman Monclou, Juan Carlos Montejo-González, Neus Montserrat, María Mora Aznar, Pedro Moral-Parras, Dulce Morales, Sara Guadalupe Moreno Cano, David Mosquera Rodríguez, Rosana Muñoz-Bermúdez, José María Nicolás, Ramon Nogue Bou, Rafaela Nogueras Salinas, Marta Ocón, Ana Ortega, Sergio Ossa, Pablo Pagliarani, Anna Parera Pous, Francisco Parrilla, Leire Pérez Bastida, Purificación Pérez, Gloria Pérez Planelles, Eva Pérez Rubio, David Pestaña Laguna, Àngels Piñol-Tena, Javier Prados, Andrés Pujol, Núria Ramon Coll, Gloria Renedo Sanchez-Giron, Ferran Roche-Campo, Laura Rodriguez, Felipe Rodríguez de Castro, Silvia Rodríguez, Covadonga Rodríguez Ruiz, Jorge Rubio, Alberto Rubio López, Miriam Ruiz Miralles, Pablo Ryan Murúa, Eva Saborido Paz, Ana Salazar Degracia, Miguel Sanchez, Ana Sánchez, Bitor Santacoloma, Maria Teresa Sariñena, Marta Segura Pensado, Lidia Serra, Mireia Serra-Fortuny, Ainhoa Serrano Lázaro, Lluís Servià, Laura Soliva, Carla Speziale, Daniel Tognetti, Adrián Tormos, Mateu Torres, Sandra Trefler, Javier Trujillano, Alejandro Úbeda, Luis Urrelo-Cerrón, Estela Val, Luis Valdivia Ruiz, Montserrat Vallverdú, Maria Van der Hofstadt Martin-Montalvo, Sabela Vara Adrio, Nil Vázquez, Javier Vengoechea, Pablo Vidal Cortes, Clara Vilà-Vilardel, Judit Vilanova, Tatiana Villada Warrington, Hua Yang, Minlan Yang, Ana Zapatero

**Affiliations:** 1 Centro de Investigación Biomedica En Red – Enfermedades Respiratorias (CIBERES), Barcelona, Spain; 2 Institut d’Investigacions August Pi i Sunyer (IDIBAPS), Universitat de Barcelona, Barcelona, Spain; 3 Intensive Care Department, Hospital Universitari Vall d’Hebron, Vall d’Hebron Institut de Recerca, Barcelona, Spain; 4 Barcelona Supercomputing Center (BSC), Barcelona, Spain; 5 Hospital Universitario de Getafe, Universidad Europea, Madrid, Spain; 6 Critical Care Department, Hospital Joan XXIII, Tarragona, Spain; 7 Translational Research in Respiratory Medicine, Respiratory Department, Hospital Universitari Aranu de Vilanova and Santa Maria, IRBLleida, Lleida, Spain; 8 Hospital Universitario Río Hortega de Valladolid, Valladolid, Spain; 9 Instituto de Investigación Biomédica de Salamanca (IBSAL), Gerencia Regional de Salud de Castilla y León, Salamanca, Spain; 10 Pulmonary Department, University and Polytechnic Hospital La Fe, Valencia, Spain; 11 Intensive Care Clinical Unit, Hospital Universitario Virgen de Rocío, Sevilla, Spain; 12 Servicio de Medicina Intensiva, Hospital Universitario La Paz, IdiPAZ, Madrid, Spain; 13 Hospital Santa Maria, IRBLleida, Lleida, Spain; 14 Hospital Universitario HM Montepríncipe, Universidad San Pablo-CEU, Madrid, Spain; 15 Servicio de Medicina Intensiva, Hospital Universitario Ramón y Cajal, Madrid, Spain; 16 Department of Intensive Care Medicine, Hospital Clínico Universitario Valladolid, Valladolid, Spain; 17 Critical Care Department, Hospital Universitari Arnau de Vilanova, IRBLleida, Lleida, Spain; 18 Hospital Álvaro Cunqueiro, Vigo, Spain; 19 Intensive Care Unit, Hospital Clínico y Universitario de Valencia, Valencia, Spain; 20 Department of Intensive Care Medicine, Hospital Universitario, 12 de Octubre, Madrid, Spain; 21 Department of Medicine, CHUS, Complejo Hospitalario Universitario de Santiago, Santiago de Compostela, Spain; 22 Department of Intensive Care, Hospital Universitari de Bellvitge, L’Hospitalet de Llobregat, Barcelona, Spain; 23 Bellvitge Biomedical Research Institute (IDIBELL), L’Hospitalet de Llobregat, Barcelona, Spain; 24 Hospital de Mataró de Barcelona, Barcelona, Spain; 25 Department of Medicine, Universitat Autònoma de Barcelona (UAB), Barcelona, Spain; 26 Critical Care Department, Corporació Sanitària Parc Taulí, Sabadell, Barcelona, Spain; 27 Departamento Medicina Facultad Medicina, Universidad de Cádiz, Hospital Universitario de Jerez, Jerez de la Frontera, Spain; 28 Unidad de Cuidados Intensivos, Hospital San Pedro de Alcántara, Cáceres, Spain; 29 Intensive Care Unit, Hospital San Juan de Dios del Aljarafe, Sevilla, Spain; 30 Intensive Care Clinical Unit, Hospital Universitario Virgen Macarena, Seville, Spain; 31 Hospital General Universitario Gregorio Marañón, Madrid, Spain; 32 Pulmonary and Critical Care Division, Emergency Department, Clínica Sagrada Família, Barcelona, Spain; 33 Intensive Care Department, Hospital Nuestra Señora de Gracia, Zaragoza, Spain; 34 Unidad de Medicina Intensiva, Hospital Universitario Virgen de Valme, Sevilla, Spain; 35 Critical Care Department, Hospital del Mar-IMIM, Barcelona, Spain; 36 Department of Intensive Medicine, Hospital Universitario Infanta Leonor, Madrid, Spain; 37 Critical Care Department, Hospital Universitario Lucus Augusti, Lugo, Spain; 38 Complejo Asistencial Universitario de Palencia, Palencia, Spain; 39 Departamento de Biología Funcional, Instituto Universitario de Oncología del Principado de Asturias, Universidad de Oviedo, Oviedo, Spain; 40 Instituto de Investigación Sanitaria del Principado de Asturias, Hospital Central de Asturias, Oviedo, Spain; 41 Servei de Medicina Intensiva, Hospital Universitari Son Espases, Palma de Mallorca, Illes Balears, Spain; 42 Servicio de Medicina Intensiva, Hospital Universitario Marqués de Valdecilla, Santander, Spain; 43 UGC-Medicina Intensiva, Hospital Universitario Reina Sofia, Instituto Maimonides IMIBIC, Córdoba, Spain; 44 Servei de medicina intensiva, Hospital Universitari Germans Trias, Badalona, Spain; 45 Hospital Verge de La Cinta, Tortosa, Tarragona, Spain; 46 Hospital de Sant Joan d’Alacant, Alacant, Spain; 47 Servicio de medicina intensiva, Hospital Universitario y Politécnico La Fe, Valencia, Spain; 48 Intensive Care Unit, Hospital Son Llàtzer, Palma de Mallorca, Illes Balears, Spain; 49 Critical Care Department, Hospital Dr. Negrín Gran Canaria, Las Palmas, Gran Canaria, Spain; 50 Intensive Care Unit, Hospital Universitario La Princesa, Madrid, Spain; 51 Critical Care Department, Hospital Universitario Río Hortega de Valladolid, Valladolid, Spain; 52 Servicio de Medicina Intensiva, Hospital Universitario Mútua de Terrassa, Terrassa, Barcelona, Spain; 53 Servei de Pneumologia i Al·lèrgia Respiratòria, Hospital Clínic, Villarroel 170, Esc 6/8 Planta 2, 08036 Barcelona, Spain

## Correction to: Crit Care (2021) 25:331 10.1186/s13054-021-03727-x

Following publication of the original article [1], the authors identified an error in the Funding section.

The updated Funding section is given below and the changes have been highlighted in **bold typeface**.

**Funding**

Financial support was provided by the Instituto de Salud Carlos III de Madrid (COV20/00110, ISCIII), **Fondo Europeo de Desarrollo Regional (FEDER), "Una manera de hacer Europa",** and by the Centro de Investigación Biomedica En Red – Enfermedades Respiratorias (CIBERES). DdGC has received financial support from Instituto de Salud Carlos III (Miguel Servet 2020: CP20/00041), co-funded by European Social Fund (ESF)/”Investing in your future”.

The original article [1] has been corrected.
